# The Importance of Biological Oscillators for Hypothalamic-Pituitary-Adrenal Activity and Tissue Glucocorticoid Response: Coordinating Stress and Neurobehavioural Adaptation

**DOI:** 10.1111/jne.12247

**Published:** 2015-05-26

**Authors:** G M Russell, K Kalafatakis, S L Lightman

**Affiliations:** Henry Wellcome Laboratories of Integrative Neuroscience and Endocrinology, School of Clinical Sciences, Faculty of Medicine and Dentistry, University of BristolBristol, UK

**Keywords:** pulsatility, hypothalamic–pituitary–adrenal axis, glucocorticoids, ultradian rhythm

## Abstract

The hypothalamic-pituitary-adrenal (HPA) axis is critical for life. It has a circadian rhythm that anticipates the metabolic, immunoregulatory and cognitive needs of the active portion of the day, and retains an ability to react rapidly to perceived stressful stimuli. The circadian variation in glucocorticoids is very ‘noisy’ because it is made up from an underlying approximately hourly ultradian rhythm of glucocorticoid pulses, which increase in amplitude at the peak of circadian secretion. We have shown that these pulses emerge as a consequence of the feedforward–feedback relationship between the actions of corticotrophin hormone (ACTH) on the adrenal cortex and of endogenous glucocorticoids on pituitary corticotrophs. The adrenal gland itself has adapted to respond preferentially to a digital signal of ACTH and has its own feedforward–feedback system that effectively amplifies the pulsatile characteristics of the incoming signal. Glucocorticoid receptor signalling in the body is also adapted to respond in a tissue-specific manner to oscillating signals of glucocorticoids, and gene transcriptional and behavioural responses depend on the pattern (i.e. constant or pulsatile) of glucocorticoid presentation. During major stressful activation of the HPA, there is a marked remodelling of the pituitary–adrenal interaction. The link between ACTH and glucocorticoid pulses is maintained, although there is a massive increase in the adrenal responsiveness to the ACTH signals.

## Introduction

It is 65 years since Hans Selye (1) demonstrated the critical role of the hypothalamic-pituitary-adrenal (HPA) axis for life and the powerful effects of its response to stress. Subsequently, glucocorticoids, which are the primary output of this axis, were shown to have a remarkably wide spectrum of activities in multiple mammalian tissues. They have a pivotal role in the maintenance of internal homeostatic processes (2) and their regulatory effects show temporal, tissue and cell specificity. Furthermore, the response to glucocorticoids will vary depending on the prior state of the body, which, in terms of central nervous system (CNS) effects, will include whether they are released on a basal or stressed background and whether conditions are pathological or normal. Glucocorticoids modulate cytotoxic (neuronal/glial death, oxidative stress) and chemotactic phenomena characteristic of neuroinflammatory processes (3), and alter neuronal metabolic homeostasis (glucose utilisation, ATP production) (4) and viability (5). Moreover, at a supracellular circuit level, glucocorticoids interact with the major neurotransmitters and many secondary neuropeptidergic systems, effectively influencing different aspects of cognitive phenotypes, such as learning ability, performance, emotional perception and overall mood (6). The output of these systems depends not only on the physiological or pathological status of the animal, but also on the timing of endogenous glucocorticoid pulses (7). During stressful conditions, glucocorticoids play a key role in orchestrating the short-term autonomic and behavioural defences against the stressor, as well as the long-term physiological responses that provide adaptation for future confrontation to similar insults (8). In this context, glucocorticoids regulate the effectiveness of many CNS outputs modulating immunological, metabolic and cognitive processes (9).

The principal endogenous glucocorticoids are cortisol (mammals including man) and corticosterone (the majority of rodents and birds), which we shall collectively refer to as CORT. CORT is characterised by its circadian pattern of basal secretion from the adrenal glands, with the highest levels being seen just prior to the start of the active cycle (i.e. just prior to awakening), followed by a gradual decline, reaching nadir levels during the inactive phase. This daily rhythm is under central regulation by corticotrophin-releasing hormone (CRH) and arginine vasopressin (AVP) from the paraventricular nucleus (PVN) of the hypothalamus. The PVN receives a powerful input from the suprachiasmatic nucleus (SCN) (10,11), predominately via projections from the subparaventricular zone. The SCN is the major co-ordinator of the whole body's circadian activities and regulates the CRH and AVP secretion from the PVN by providing an inhibitory effect during the inactive phase of the cycle in addition to the feedback inhibition from circulating CORT (12,13). Additionally, the PVN receives internal and external inputs from the limbic system and brain stem; these inputs are responsible for mounting appropriate stress responsiveness to cognitive, emotional and physiological stressors (14,15).

After synthesis, CRH and AVP are released into the hypophyseal portal system via axonal projections to the median eminence. Both CRH and AVP are transported via the vasculature to the pituitary where they activate pituitary corticotrophs. Here, they stimulate the release of corticotrophin (ACTH), which in turn is released into the general systemic circulation and stimulates the adrenal cortex to produce CORT. After *de novo* synthesis and release into the systemic circulation, CORT is able to act at target tissues to exert metabolic, cardiovascular, immunological and cognitive responses in response to the relevant inputs (14). At physiological levels, CORT auto-restricts its further production by forming negative-feedback loops at the levels of pituitary, the hypothalamic PVN and hippocampus to inhibit further ACTH and CORT release (14,16).

Splanchnic nerve innervation of the adrenal glands also contributes to the circadian rhythm. The adrenal glands receive autonomic (sympathetic) innervation that lies under the influence of neuronal projections of the autonomic portion of the PVN and, consequently, the SCN to the spinal cord (17). The splanchnic nerve alters adrenal sensitivity, transection increases CORT secretion in the inactive phase but has no effect in the active phase, suggesting that the sympathetic nervous system exerts an inhibitory effect on CORT during the circadian nadir (18). The splanchnic nerve also mediates a light-induced mechanism that can alter adrenal clock gene function and CORT synthesis. This phenomena acts during both the subjective day and night, with the irradiance threshold being greater during the subjective day. This suggests that photic signals may reach the adrenal gland via a SCN independent mechanism and be involved in the temporal physiology of CORTs (19,20). Additionally, the adrenal gland itself can exert internal homeostatic control via an autonomous clock that influences ACTH sensitivity and adrenal steroidogenesis (21,22).

The diurnal pattern of peak and trough CORT secretion is considerably more pronounced than the circadian pattern of ACTH. Studies examining 24-h cortisol and ACTH profiles of healthy individuals reveal a four- to six-fold difference between the circadian peak and trough amplitudes of CORT, whereas the corresponding ACTH difference is approximately two- to three-fold. The characteristics of these 24-h profiles alter with age (trough CORT and ACTH levels are higher in elderly people) (23) or in response to pathological conditions, including major depression, Alzheimer's disease and Parkinson's disease (23–25).

The circadian variation of cortisol also interacts with several other important biological oscillations, such as activity, body temperature (26) and the transcriptional activity of many glucocorticoid responsive genes. These genes include tryptophan hydroxylase in the raphe nucleus of the brainstem, which has a circadian rhythmicity that can be abolished by exogenous steroids (27) and is a gene that is implicated in regulation of affect, activity and temperature.

The classic transcriptional (genomic) effects of CORT are relatively slow and are mediated by activation of its cognate intracellular nuclear receptors, the glucocorticoid receptor (GR) and the mineralocorticoid receptor (MR) (2,28). MRs have approximately five- to ten-fold higher affinity for CORT compared to GRs (29). Nevertheless, glucocorticoids can also exert rapid, nongenomic effects by acting at the level of cellular membranes (30,31); these effects may include the classical nuclear receptors (32), as well as membrane bound variants (33). The latter have a lower affinity for glucocorticoids than the classical nuclear receptors (34). As a result, although nuclear MRs are almost constantly occupied even at low concentrations, nuclear GR and membrane associated MRs and GRs are effectively only occupied and activated whenever CORT levels rise sufficiently, with prime examples being towards the circadian peak or under stressful conditions that up-regulate HPA activity (2).

The distribution of these receptors is tissue-specific, providing an additional mechanism for regulatory control, with GRs being present throughout the brain and periphery and MRs having a more limited localisation; they are mainly present in cardiovascular tissues, liver and kidneys, as well as in most corticolimbic regions of the brain, including those involved in HPA axis regulation (2). Hippocampus, basal ganglia, lateral septum and medial amygdala neurones present a high MR : GR ratio (35) and, because MR remain occupied even during nadir levels of CORT, these remain under constant, nuclear MR-dependent, genomic regulation of glucocorticoids, which involve stabilising, homeostatic events (9). When CORT levels rise, these brain areas become additionally susceptible to the membrane MR-dependent, nongenomic, rapid effects of glucocorticoids (36), which prepare the behavioural response of the individual to a stressor by enhancing processes such as synaptic plasticity (at a cellular level) (37) and, collectively, predictability, decision-making, selective attention and risk assessment (at a cognitive level) (38). Additionally, under high CORT levels, brain regions such as hypothalamic PVN, lateral amygdala, cerebellum and dorsomedial prefrontal cortex (PFC) with a low MR : GR ratio (35) become susceptible to the genomic (late) and nongenomic (rapid) GR-dependent effects, which, on the one hand, counteract membrane MR-coordinated actions by promoting the attenuation or even termination of any initiated stress response and, on the other hand, enhance the long-lasting, neurobehavioural adaptive mechanisms, such as strategic planning, memory storage and consolidation (38). Other brain areas, such as lateral geniculate, orbitofrontal and dorsolateral PFC, whose MR : GR ratio is approximately 1 (35), may also contribute to these processes. Glial populations tend to express both kinds of GRs (39), and thus may differentially respond to glucocorticoid-related effects based on their concentration and the temporal pattern of exposure.

The physiology underlying nongenomic, rapid glucocorticoid effects has been linked to various second messenger cellular systems under different conditions. For example, the binding of pharmacological doses of CORT to GR stimulated phosphatidylinositol 3-kinase and protein kinase Akt, leading to acute, cardioprotective endothelial NO synthase activation and nitric oxide-dependent vasorelaxation in the heart of rat models of myocardial infarction (40). At the level of the prefrontal cortex, increased CORT impairs working memory and enhances memory consolidation via membrane GR activation, which promotes noradrenergic-dependent activation of adenylate cyclase, leading to increased intracellular cAMP concentrations and cAMP-dependent protein kinase A activation (41). On the other hand, nongenomic MR effects include increasing intracellular calcium concentrations and activating protein kinases (A, C, mitogen-activated) and Rac1 (a small G protein) (42).

## Ultradian rhythm

**Fig. 1:**
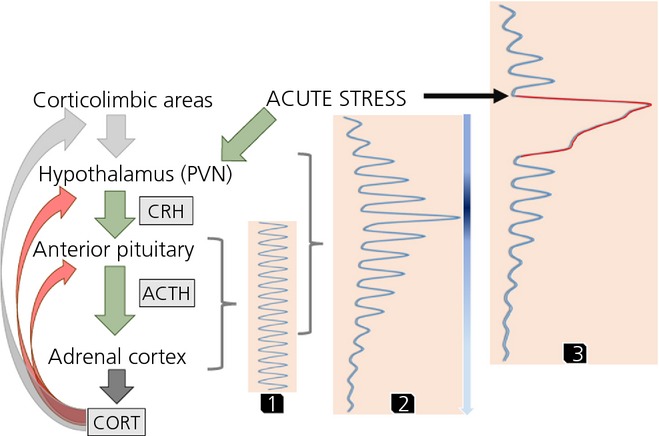
The principal regulatory mechanisms that underlie hypothalamic-pituitary-adrenal (HPA) activity. Corticotrophin-releasing hormone (CRH) released from the paraventricular nucleus (PVN) of the hypothalamus reaches the anterior pituitary through the hypophyseal portal circulation, and stimulates corticotrophs to release corticotrophin (ACTH), which in turn reaches the adrenal gland through the systemic circulation and promotes the cortical synthesis and secretion of glucocorticoids (CORT). CORT, in turn, exerts an auto-inhibitory effect on their production by acting on at least three different levels: anterior pituitary, hypothalamus and hippocampus (displayed as part of corticolimbic system). CORT also affects extensive corticolimbic regions of the brain, which in turn modulate, primarily via indirect projections, the mode of HPA axis activity. (1) CORT pulsatility emerges as a consequence of the feedforward–feedback with a built-in delays relationship between the actions of ACTH on the adrenal cortex and endogenous CORT on the pituitary corticotrophs. (2) Physiological CRH drive creates the variability in the amplitude and duration of each CORT pulse throughout the day, which enables the gradual increase of CORT levels during the most active parts of the day (darker parts of the blue arrow) and their gradual fall during the less active parts of the day (lighter parts of the blue arrow). (3) An acute stressor, leading to a substantial raise in the hypothalamic CRH secretion, results in increased CORT levels characterised by a dampened oscillatory profile, and eventually resets the phase of the ultradian rhythm. Green arrows, stimulatory effect; red arrows, inhibitory effect; grey arrows, mixed effect.

The circadian variation seen in CORT's natural rhythm is made up of underlying discrete pulses of HPA activity, a rapid and dynamic ultradian rhythm (Fig.1) (43). This was previously interpreted as noise in the system, primarily because it was notoriously difficult to study basal unstressed conditions. As a result of developments in automated blood sampling (44,45) and mathematical modelling techniques, which allow accurate interpretation of secretory dynamics, such as deconvolution analysis (43), it is possible to study basal HPA activity in more depth. This pulsatile pattern of secretion is produced at an approximately frequency of 60–90 min (43,46,47) with an increase in pulse amplitude and frequency corresponding to the circadian peak of secretion (43). This has been documented in all mammalian species studied (45,48,49), including humans (43). The actual pattern of secretion is highly individual and marked variations are seen according to genetic background, sex hormones, neonatal epigenetic programming effects, environmental stressors and changes associated with age (50–53).

The origin of the ultradian rhythm has been subject to many hypotheses, although it has always been assumed to result from some elusive hypothalamic pulse generator. Recently, however, it has become clear that, unlike the circadian rhythm, ultradian pulsatility is not under central regulation from the SCN because, as in SCN-lesioned rodents, the ultradian rhythm persists despite a loss in circadian rhythmicity (54). The central hypothalamic ‘pulse generator’ hypothesis (55) envisaged that CRH has pulsatile characteristics (55–57) and that these pulses of CRH must drive ACTH (58) and glucocorticoid pulsatility resulting in the ultradian rhythm. However, there are a few caveats; first, there is a mismatch in pulse frequency. In rodents, CRH pulse frequency is significantly higher at three pulses per hour (55) in comparison to hourly ACTH (59) and corticosterone pulses (45). Additionally, ACTH and glucocorticoid pulsatility show autonomy from the hypothalamus. In sheep, pituitary-adrenal pulsatility continues after hypothalamic disconnection from the pituitary gland (60), suggesting a subhypothalamic origin.

The breakthrough in our understanding of the origin of ultradian pulsatility came from mathematical modelling (61), which created predictions that were later supported by *in vivo* experimental work. This modelling was based on the previously accepted understanding that (i) there is a delay in CORT secretion because of the requirement for its *de novo* synthesis and (ii) this CORT can induce a rapid, nongenomic negative-feedback at the level of the pituitary corticotrophs to inhibit CRH induced ACTH secretion (62,63). This creates a feedforward–feedback system with built-in delays that, for simple mathematical reasons, has to oscillate. We have used mathematical modelling to describe how the constant CRH drive will result in activation of the subhypothalamic-pituitary-adrenal oscillator with oscillating levels of both ACTH and CORT at a physiological frequency. However, if the magnitude of CRH drive decreases significantly, as occurs at night in man and in the morning in the rodent, oscillatory activity will cease, whereas, in contrast, under very high CRH levels, perhaps after an acute stressor, oscillatory activity will be damped. A further prediction from our mathematical models is that a stressor can induce phase shifts in the ultradian pattern, in effect acting as a resetting mechanism for the phase of the ultradian rhythm, and that the ability to respond to perturbations would be much greater under oscillatory rather than equilibrium conditions (64).

This model of a subhypothalamic origin of pituitary-adrenal pulsatility was tested *in vivo* by examining endogenous ACTH and corticosterone responses in freely running male Sprague–Dawley rats under different rates of constant CRH infusions at the circadian trough when endogenous CRH drive was very low and there was minimal secretion of endogenous ACTH and corticosterone. As predicted by the mathematical model (61), constant CRH infusion induced an ultradian pattern of ACTH and corticosterone secretion with consistent pulse frequency and amplitudes for the duration of the infusion and with ACTH oscillations preceding CORT ones. This pulsatile response was dose-dependent and, when high levels of CRH were infused, the pattern was disrupted with a large pulse of ACTH and CORT followed by a dampening of CORT oscillations. In addition, in comparison with control rodents at the circadian peak when endogenous CRH activity is maximal, frequency analysis revealed no significant difference between these endogenous oscillations and the oscillations induced by constant CRH infusion. These data confirm that pulses emerge as a consequence of the feedforward–feedback relationship between the actions of ACTH on the adrenal cortex and endogenous glucocorticoids on the pituitary corticotrophs (61,65). Whether the pulsatility of hypothalamic CRH secretion could impact on this subhypothalamic system remains unknown.

The adrenal gland itself preferentially responds to an oscillatory ACTH signal; in methylprednisolone (a synthetic glucocorticoid) suppressed rodents, pulsatile ACTH infusions result in pulsatile corticosterone secretion. When a constant infusion of ACTH is given at the same total dose, there is no adrenal response at all (66). We have now demonstrated that the adrenal gland also has its own internal feedforward–feedback system that is able to effectively amplify the response to rapid changes of ACTH inherent in the pulsatile characteristics of the incoming signal. In the rat, a pulse of ACTH induces a pulse of steroidogenic transcription (67,68); specifically, steroidogenic acute regulatory protein (StAR), a key steroidogenic gene that is a rate-limiting step in the production of steroid hormones promoting intra-mitochondrial cholesterol translocation for subsequent steroidogenesis and increased CORT (69), shows a rapid rise in heteronuclear RNA (hnRNA) levels within 15 min (68), and returns to basal levels by 30 min. Similar responses were seen in protein phosphorylation for CREB (68), involved in the regulation of StAR (70), and hnRNA levels in melanocortin 2 receptor accessory protein (68), involved in the level and activity of the melanocortin receptor and thus the cells’ responsiveness to ACTH (71) This suggests that the intra-adrenal dynamics of response to each pulse of ACTH effectively sensitises its responsiveness in the short term, an effect that appears to be lost with constant infusion.

## Glucocorticoid-induced gene transcription pulsatility

**Fig. 2:**
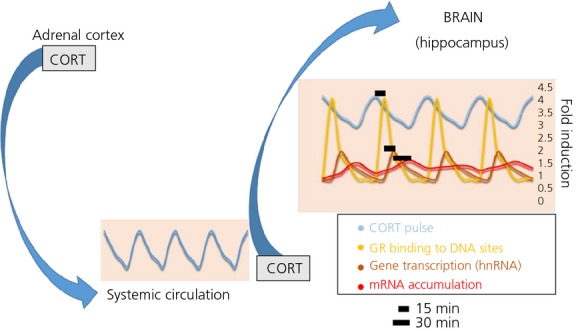
Glucocorticoids (CORT) are secreted in a pulsatile manner from the adrenal cortex to systemic circulation, where they predominantly interact with corticosteroid-binding globulin and, to a lesser extent, with albumin. Only the free fraction of CORT (approximately 5%) is biologically active, and this fraction oscillates synchronously between the blood and the brain (blue curves). In the latter, and particularly within the hippocampus, free CORT pulsatility induces ‘gene pulsing’; 15 min after a CORT pulse, GR maximises its translocation to the cellular nucleus and its binding to corresponding DNA sites (yellow curve) and, approximately 15 min later, CORT-sensitive genes such as period 1 reach a peak in their transcriptional levels [heteronuclear RNA (hnRNA) levels]. Thirty minutes later, corresponding mRNA accumulation also reaches its maximum. GR, glucocorticoid receptor.

CORT in the circulation is bound predominantly to corticosteroid-binding globulin (CBG) and, to a lesser extent, to albumin. It is only the free fraction of CORT that is active, and this comprises only approximately 5% of total CORT under basal (nonstressed) trough levels (72). The biological relevance of pulsatile CORT secretion would only be significant, therefore, if it also resulted in pulsatile concentrations of free hormone. Because CBG is saturated at relatively low CORT concentrations, including the basal (nonstressed) levels found at the circadian peak of CORT secretion (72) and because, similar to CORT, CBG exhibits diurnal variation (73), resulting in a higher proportion of free CORT during the circadian peak, CBG actually accentuates free CORTs diurnal profile (74,75). Any pulse above the saturation threshold will result in a disproportionately high pulse of free and thus active CORT. To establish whether or not this was indeed the case, we performed *in vivo* microdialysis studies that confirmed synchronous total blood and hippocampal free CORT pulses (76,77). The next question is whether these pulses of tissue CORT are paralleled by pulses of glucocorticoid receptor binding and gene transcription. This has now been confirmed both *in vitro* and *in vivo*, with evidence that different patterns of glucocorticoid presentation have differential effects on gene regulation, and that these effects are highly specific for the endogenous glucocorticoids (CORT) only. Indeed, oscillations in CORT induce a phenomenon known as ‘gene pulsing’ (Fig.2) (78,79); as CORT levels rise during an endogenous pulse, CORT binds and activates GR, which is translocated into the nucleus, dimerises and interacts with glucocorticoid response elements (GRE) on deoxyribonucleic acid (DNA) to initiate transcription. There is rapid cycling of GR and transcription factors on and off chromatin, and, as CORT levels fall, GR is dissociated from its substrate and released into the nucleoplasm waiting for the next CORT pulse when it can be reactivated, rapidly responding to the next pulse. Under constant presentation of CORT (as seen in glucocorticoid-based therapeutics), the mode of genomic responses changes, with the transcription levels of many glucocorticoid responsive genes constantly rising, exhibiting a very different pattern of gene transcription (78). In addition to the classical GRE-dependent interactions, GR can interact with a large cohort of other transcription factors via DNA-binding mechanisms influencing DNA accessibility, chromatin remodelling and transcriptional regulation (80), as well as DNA independent binding mechanisms via protein–protein interactions (81,82). GR transcriptional control is therefore dynamic, diverse and reliant on its co-operative partners. This helps to explain the wide-ranging and variable GR cell and tissue specific effects that are observed (83).

In a similar manner, different patterns of glucocorticoid presentation exert different neuronal and behavioural responses. Indeed, the c-*fos* mRNA response to the neural activation by noise stress is markedly altered in the amygdala, hippocampus and hypothalamic PVN depending not only on the pattern of presentation (constant versus pulsatile), but also on the phase within each pulse. During the rising phase of the pulse, an exaggerated noise stress response was observed in comparison to the falling phase (84). Quite remarkably, this alternation of c-*fos* mRNA levels was in parallel to differences in the behavioural responses observed in response to this stressor. The same pattern of pulse phase dependency has been observed in aggressive and novelty behaviour, as well as rapid HPA negative-feedback (45,62,84,85).

The rapidity of these neurochemical and behavioural responses to pulses of CORT implies the involvement of nongenomic biological events in addition to the more classical phasic GR and MR co-ordinated effects. There is growing evidence that this is the case and we shall outline the evidence on the importance of pulsatility for cognitive responses further below.

## Glucocorticoid ultradian rhythmicity and stress activation

During major stress-mediated activation of the HPA axis, there is a marked increase in CORT levels, initially accompanied by an elevation of ACTH levels, which then return to normal or subnormal levels despite CORT levels remaining elevated (86,87). Many explanations have been suggested for this phenomenon, including alternative mechanisms for CORT production mediated by autonomic projections (88,89) and pro-inflammatory cytokines (90,91). Another possibility is that the initial high levels of ACTH lead to altered adrenal sensitivity to subsequent ACTH stimulation. Our preliminary unpublished data suggest that a marked remodelling of the pituitary–adrenal interaction takes place during a major stressful activation. The synchrony between ACTH and CORT pulses is maintained, although this is combined with a massive increase in adrenal responsiveness to the ACTH signals (92).

## Impact of rapidly changing levels of glucocorticoids on cognition

Glucocorticoids are key regulators of learning and memory (emotional, social and stress-related) for which the neuroanatomical bases lie in the corticolimbic areas and primarily the hippocampus. Within these systems, glucocorticoids interact at multiple levels, resulting in structural and frequently opposing or bidirectional functional consequences (93). At the synaptic level, glucocorticoids modulate the (presynaptic) activity and postsynaptic responses to glutamatergic and GABAergic neurotransmissions, which are crucially involved in the molecular phenomena of long-term potentiation (LTP) and long-term depression underlying learning. At a cellular level, glucocorticoids influence the electrical properties of neuronal activity (94) and the turnover of dendritic spines, both important features for effective trans-neuronal communication (95). Moreover, glucocorticoids interact with the noradrenergic and cholinergic circuits that innervate the hippocampus and amygdala, and conditionally affect memory formation, as well as with secondary neuropeptidergic systems including the endocannabinoids (96), also important in regulating these behavioural adaptations. The variable nature of these effects is determined by the phase of the glucocorticoid ultradian pulse, the individual's systemic glucocorticoid concentrations, the timing of the stress in relationship to learning/cognition and the type of learning episode (97,98). Acute increases in glucocorticoids just prior to/during/immediately post learning can promote processes such as memory formation, consolidation and recall of emotionally arousing stimuli (98–100). However, if the stress is temporally well before the learning and consequently genomic glucocorticoid actions are present, this can impede memory processes (101). In addition, chronically raised glucocorticoid concentrations can impair spatial and retrieval of memory (98,102).

Acute stress/high levels of glucocorticoids increase glutamate release primarily from neuronal (and secondary from glial) populations in hippocampus and PFC by increasing the number or the probability of vesicular exocytosis at the presynaptic level via a rapid nongenomic MR effect (103). This is followed by increased translocation of NMDA and, independently, AMPA receptors from intracellular pools to the postsynaptic plasma membrane (104). Moreover, acute stress enhances a NMDA receptor-independent form of LTP by mobilising calcium-permeable AMPA receptors in a glucocorticoid-dependent manner (105). The duration of this MR-dependent up-regulation of glutaminergic neurotransmission is brain-region specific, being short-lasting at the hippocampus and long-lasting in the basolateral amygdala, where subsequent acute stressful insults lead to a GR-dependent down-regulation of glutaminergic stimulation (106). During chronic stress, glutaminergic neurotransmission remains constant in the hippocampus, whereas it gradually decreases in the PFC. There is also a PFC-specific down-regulation of NMDA and AMPA receptors as a result of disrupted receptor trafficking and/or altered degradation or synthesis. Glucocorticoids also affect glutamate clearance from the glial cells through glutamate transporters primarily expressed in these cellular populations; acute stress increases, whereas chronic stress decreases, glutamate uptake (clearance) and metabolism in the frontal cortex and hippocampus (107). Concerning GABAergic neurotransmission within corticolimbic areas, glucocorticoids enhance neurotransmission under low and high concentrations by increasing the binding affinity of GABA_A_ receptors, whereas basal levels reduce neurotransmission (108).

**Fig. 3:**
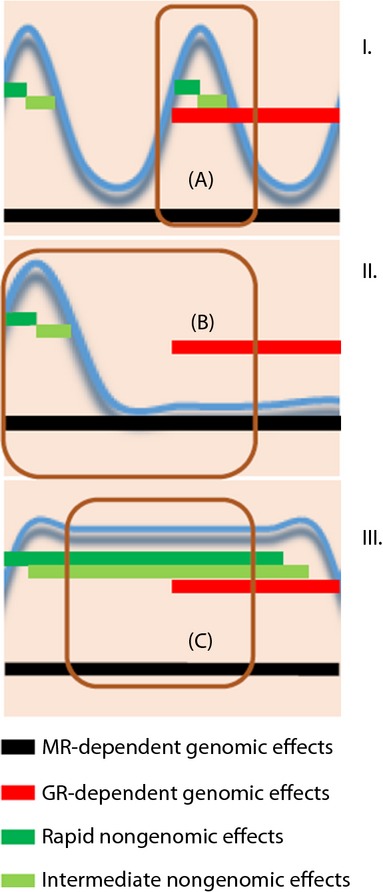
Theoretical approach of the varying interactions among genomic and nongenomic glucocorticoid effects under differential patterns of exposure; (I) glucocorticoid pulses (blue curve) characterised by physiological interpulse intervals (mean duration of approximately 90 min) lead to a short-lasting association between their rapid/intermediate and delayed effects (a), whereas (II) glucocorticoid pulses (blue curve) characterised by prolonged interpulse intervals (over 4–5 h) lead to a complete dissociation between their rapid/intermediate and delayed effects (b). (III) Finally, acute stress conditions (blue curve = prolonged high glucocorticoid levels, diminished or no pulsatility) could result in a prolonged interplay between genomic and nongenomic effects (c). Different patterns of glucocorticoid exposure have been related to changing phenotypes in various neuronal functions, such as long-term potentiation (LTP) induction. Black lines, period of delayed/genomic mineralocorticoid receptor (MR)-dependent effects; red lines, period of delayed/genomic GR-dependent effects; dark green lines, rapid, nongenomic effects; light green lines, intermediate, nongenomic effects. GR, glucocorticoid receptor.

In relation to the wide spectrum of glucocorticoid actions, and under different physiological or pathological circumstances, pulsatility offers a means of temporally dissociating or combining the MR- from the GR-dependent actions (Fig.3). This temporal dissociation of the different glucocorticoid-responsive receptors provides the potential for pulsatility to increase the diversity of glucocorticoid-coordinated responses, which will depend on the fluctuating concentrations of CORT and the duration of secretory pulse derived high levels within the brain, which, in turn, will change depending on the physiological or pathological state. These glucocorticoid responses are temporally dependent and frequently opposing, leading to opposing biological phenomena; glucocorticoids inhibit pro-inflammatory cytokines under basal conditions or acute stress (less than 1 h prior to stress), whereas acute glucocorticoid exposure greater than 1 h prior to stress along with chronic stress will enhance neuroinflammatory responses (5,109,110). Other examples include the brain region-, metabolic state- and developmental stage-dependent neuroprotective effects of physiological levels of glucocorticoids versus their neurotoxic effects under increased concentrations (5) or the brain region-specific enhancement versus attenuation of glutaminergic neurotransmission under acute versus chronic stress conditions, respectively (105). On the other hand, by periodically synchronising MR and GR activations, pulsatility achieves a further extension in the diversity of glucocorticoid-related regulatory capabilities within the CNS because nuclear MRs and GRs can form heterodimeric complexes with DNA-binding and transactivation properties different from those of the respective homodimers (111).

Returning to the effects of glucocorticoids on synaptic activity and neurotransmission, a recent elegant study (112) provides fascinating evidence for the importance of the pulsatile exposure of glutaminergic synapses to CORT in enhancing their plastic properties. The study investigated the differential impact of one versus two CORT pulses (with a 1-h interpulse interval, mimicking the normal ultradian frequency pattern) on two aspects of the physiology of glutaminergic neurotransmission, namely the synaptic mobilisation of AMPA receptors and LTP induction in cultures of hippocampal neurones and dorsal hippocampal slices from rodent brains. Interestingly, one versus two pulses of CORT resulted in opposing phenomena: a single CORT pulse significantly increased the surface diffusion and synaptic accumulation of AMPA receptors leading to increased amplitude of miniature excitatory postsynaptic currents, whereas the second pulse eliminated these effects. Moreover, although a single CORT pulse inhibited LTP induction in a genomic manner, the second pulse restored this electrophysiological synaptic feature by acting at a GR-dependent nongenomic level.

The combined assessment of all reported data concerning glucocorticoid–glutaminergic interactions in hippocampus (103–107), with an emphasis on this most recent study of Sarabdjitsingh *et al*. (112), provides a remarkable example of the temporal dissociation as well as combination between rapid and slow (MR- or GR-dependent) actions achieved by pulsatility, which offers an explanation for the vastly diverse actions of glucocorticoids. The experimentally-induced transition from no CORT influence to an increased but time-limited (20 min) CORT stimulation, mimicking an ultradian pulse, comprises an example of temporally dissociating the various functions of glucocorticoid-sensitive receptors; under these conditions, CORT promotes (i) direct, nongenomic (and thus rapid) effects, such as the increase of glutamate release (MR-dependent action), facilitation of NMDA receptor activation, and enhancement of GluA1-AMPA receptor activation and surface expression (GR-dependent action); (ii) indirect nongenomic (and thus subacute) phenomena, such as the normalising endocannabinoid-induced inhibition of glutamate release (MR-dependent action); and (iii) direct, genomic (and thus delayed) effects, such as the suppression of LTP induction (GR-dependent action) and the enhancement of the synaptic plasticity-related mitogen-activated protein kinase-ERG1 pathway (GR-dependent action). On top of this, the application of a second pulse of CORT after the initial one provides a mechanism for temporally associating glucocorticoid-sensitive receptor functions. The duration of the temporal association between CORT and its receptors is also of critical importance, as highlighted by the work of Whitehead *et al*. (105). In their study, 120 min of exposure of hippocampal slices to CORT (mimicking an acute stressor) resulted in an enhanced LTP induction as opposed to the restoration of LTP induction to control levels after the second 20-min physiological CORT pulse employed by Sarabdjitsingh *et al*. (112). How these temporal effects may contribute to the increased morbidity and mortality of patients on oral nonpulsatile CORT replacement clearly needs to be addressed.

## Disrupted glucocorticoid pulsatility and glucocorticoid resistance

The duration of tissue exposure to high CORT levels has clear biological consequences and oscillating levels of CORT allow a state of constant dynamic equilibration (113), which prevents either down-regulation of signalling processes or the abnormal prolonged activation of glucocorticoid responsive genes. The extent to which this contributes to states characterised by a sustained dysregulation of the physiological ultradian pattern, such as chronic stress, various neuropsychiatric disorders or chronic treatment with high doses of glucocorticoids (114), needs further investigation, especially those aspects associated with brain glucocorticoid resistance (115) followed by GR down-regulation and reduced GR-dependent regulatory influences (116). For example, rapid GR-dependent negative-feedback regulation of ACTH release under basal conditions or acute stress (44) may be lost in major depression, a condition accompanied by an overactive HPA axis (117). Other examples involve the reduction of immune system's sensitivity to the immunosuppressive effects of glucocorticoids during chronic psychological stress (118) or the selective down-regulation of hippocampal GRs under sustained stress in rodents and nonhuman primates (119) or after the experimental induction of viral encephalitis in rats (120). Additionally, glucocorticoid resistance has been suggested to contribute to neuropathological mechanisms related to Alzheimer's disease [another condition accompanied by an up-regulated ultradian pattern (25)] such as cortical disruption of axonal transport (121).

## Conclusions

Pulsatility is a crucial feature of glucocorticoid secretion and its regulatory effects. Only by gaining a basic understanding of its importance in normal physiology can we hope to clarify the importance of its disruption in pathological conditions. Hopefully, this will also improve our understanding and management of stress-related conditions (such as schizophrenia, anxiety and mood disorders, neurodegenerative disorders, epileptic syndromes, metabolic and vascular conditions) (122). The aim should be to develop more personalised, multivariate therapeutic approaches and prognostic indices in disease states associated with a dysregulated HPA axis, as well as improve the efficiency and attenuate any side effects of glucocorticoid-based treatments (123).
